# FISH-based karyotyping of *Pelmatohydraoligactis* (Pallas, 1766), *Hydraoxycnida* Schulze, 1914, and *H.magnipapillata* Itô, 1947 (Cnidaria, Hydrozoa)

**DOI:** 10.3897/CompCytogen.v12i2.32120

**Published:** 2018-12-20

**Authors:** Boris A. Anokhin, Valentina G. Kuznetsova

**Affiliations:** 1 Zoological Institute of Russian Academy of Sciences, St. Petersburg, 199034, Russia Zoological Institute of Russian Academy of Sciences St. Petersburg Russia

**Keywords:** *
Hydra
*, *
Pelmatohydra
*, Hydridae, karyotype, chromosomes, FISH, (TTAGGG)*_n_*, 18S rDNA, histone H3

## Abstract

An account is given of the karyotypes of *Hydramagnipapillata* Itô, 1947, *H.oxycnida* Schulze, 1914, and *Pelmatohydraoligactis* (Pallas, 1766) (Cnidaria, Hydrozoa, Hydridae). A number of different techniques were used: conventional karyotype characterization by standard staining, DAPI-banding and C-banding was complemented by the physical mapping of the ribosomal RNA (18S rDNA probe) and H3 histone genes, and the telomeric (TTAGGG)*_n_* sequence by fluorescence *in situ* hybridization (FISH). We found that the species studied had 2n = 30; constitutive heterochromatin was present in the centromeric regions of the chromosomes; the “vertebrate” telomeric (TTAGGG)*_n_* motif was located on both ends of each chromosome and no interstitial sites were detected; 18S rDNA was mapped on the largest chromosome pair in *H.magnipapillata* and on one of the largest chromosome pairs in *H.oxycnida* and *P.oligactis*; in *H.magnipapillata*, the major rRNA and H3 histone multigene families were located on the largest pair of chromosomes, on their long arms and in the centromeric areas respectively. This is the first chromosomal mapping of H3 in hydras.

## Introduction

Hydras are simple freshwater invertebrates belonging to one of the most ancient members of the animal kingdom, the phylum Cnidaria (class Hydrozoa, order Hydrida, family Hydridae). Hydras are of general interest since they display fundamental principles that underlie development, differentiation, regeneration and symbiosis (e.g. Bosch 2007, 2008, Khalturin et al. 2009, Augustin et al. 2010, Bosch et al. 2010). Some species of hydras are relatively easy animals to culture and maintain in the laboratory, then, they have been used as model organisms in many different areas of biological research, primarily in developmental biology often referred to as “evo-devo”, i.e. evolutionary developmental biology research (Slobodkin and Bossert 2001, Galliot 2012).

Without detailed knowledge of these basal metazoans, it is impossible to provide an effective comparative framework for animal evolution (Zacharias et al. 2004). Nevertheless, the species level diversity, taxonomy and phylogenetic relationships of the hydra species are far from well understood. Jankowski et al. (2008) suggested 12–15 really different hydra species, whereas Bouillon et al. (2006) reported approximately 30 valid species, and the World Register of Marine Species lists 40 species (Schuchert 2018). All hydras were originally included in the single genus *Hydra* Linnaeus, 1758. However Schulze (1914, 1917) divided hydras into three genera, *Hydra*, *Chlorohydra* Schulze, 1914, and *Pelmatohydra* Schulze, 1914, and their validity was substantiated elsewhere (e.g. Collins 2000, Stepanjants et al. 2000, Anokhin 2002).

During the past decade or so, several molecular phylogenetic studies using mitochondrial and nuclear genes shed light on the diversity within *Hydra* sensu Linnaeus, 1758 (Hemmrich et al. 2007, Kawaida et al. 2010, Martínez et al. 2010, Schwentner and Bosch 2015). The genome of one species, *Hydramagnipapillata* Itô, 1947, has been recently assembled (Chapman et al. 2010).

Chromosomes are known to be the carriers of genetic material, and chromosome changes provide the basis of speciation (White 1973). As many as 8 species from all three above-mentioned hydra genera have been karyotyped so far (Xinbai et al. 1987, Ovanesyan and Kuznetsova 1995, Anokhin et al. 1998, 2010, Anokhin and Kuznetsova 1999, Anokhin 2002, 2004, Anokhin and Nokkala 2004, Zacharias et al. 2004, Stepanjants et al. 2006, Traut et al. 2007). These species were mainly studied using conventional chromosome staining techniques, including C-banding. They were shown to have 2n = 30, almost exclusively meta/submetacentric (m/sm) chromosomes of similar size, and C-heterochromatin blocks localized in the centromeric regions of the chromosomes. Sex chromosomes were not distinguished in any species. Thus, hydras can now been considered as the group with the greatest stability in their karyotype, at least regarding the number of chromosomes. In two studies only (Traut et al. 2007, Anokhin et al. 2010), the fluorescence *in situ* hybridization (FISH) was used to characterize hydras in terms of telomeric sequences and the chromosomal distribution of the rRNA and some other genes.

Our study was aimed to add new data on hydra chromosomes studied using C-banding and FISH with probes for the “vertebrate” telomere motif (TTAGGG)*_n_*, 18S rDNA, and histone H3. We adopt here the generic hydra classification of Schulze (1914, 1917).

## Material and methods

Experiments were carried out with three species, *Hydramagnipapillata*, *H.oxycnida* Schulze, 1914, and *Pelmatohydraoligactis* (Pallas, 1766). *H.magnipapillata* (strain 105) was obtained from the Institute of Zoology, University of Kiel (Germany); *H.oxycnida* and *P.oligactis* were collected from nature (58°48'46.9"N, 29°59'02.7"E, the Oredezh river, Leningrad Province, Russia). Polyps were cultured at 18 ± 0.5 °C for a long period of time in the case of *H.magnipapillata* or for one-two weeks in the cases of *H.oxycnida* and *P.oligactis*. They were fed regularly with freshly hatched nauplii of *Artemiasalina* (Linnaeus, 1758) (Crustacea, Branchiopoda).

Different methods were tried to characterize the chromosomes of the above-mentioned species: C-banding for *H.magnipapillata* and *P.oligactis*; FISH mapping of 18S rRNA and histone H3 genes for *H.magnipapillata* and of the “vertebrate” telomere motif (TTAGGG)_n_ for *H.oxycnida* and *P.oligactis*.

Spread chromosome preparations were made from asexual polyps. Hydras were subjected to a hypoosmotic shock with 0.4% trisodium citrate for 30 min followed by fixation in ethanol and acetic acid (3:1) for 15 min. Specimens were transferred to a drop of 70% ethanol on the glass slides and dissected with needles. The cell suspension was spread by the warm air stream (37–70 °C).

In DNA isolation, 18S rDNA and (TTAGGG)*_n_* probes generation and FISH experiments we followed the protocol described in Anokhin et al. (2010). The probe for the histone H3 was PCR amplified and labeled by Rhodamine-5-dUTP (GeneCraft, Germany) using primers H3F: 5’-ATG GCT CGT ACC AAG CAG ACV GC-3’ and H3R: 5’-ATA TCC TTR GGC ATR ATR GTG AC-3’ (Huang et al. 2011).

Microscopic images were taken using a Leica DM 6000B microscope with a 100× objective, Leica DFC 345 FX camera and Leica Application Suite 3.7 software with an Image Overlay module (Leica Microsystems Wetzlar GmbH, Germany). The filter sets applied were A, L5, N21 (Leica Microsystems, Wetzlar, Germany).

## Results

**Figure 1.**
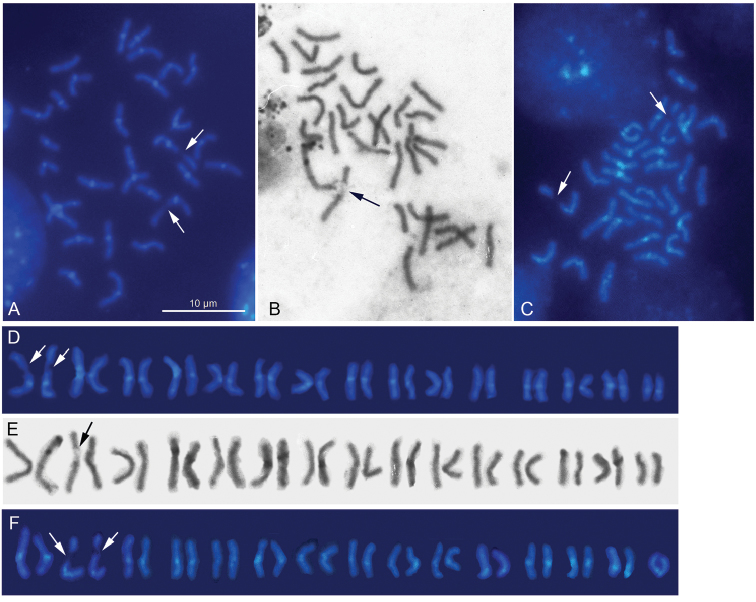
Mitotic chromosomes of *Hydramagnipapillata* after C- banding (**A**), *Hydraoxycnida* after routine staining (**B**), and *Pelmatohydraoligactis* after C- banding (**C**). C-bands are visible in the centromeric areas of the chromosomes. Karyograms of *H.magnipapillata* (**D**), *H.oxycnida* (**E**) and *P.oligactis* (**F**). Arrows indicate achromatic gaps.

**Figure 2.**
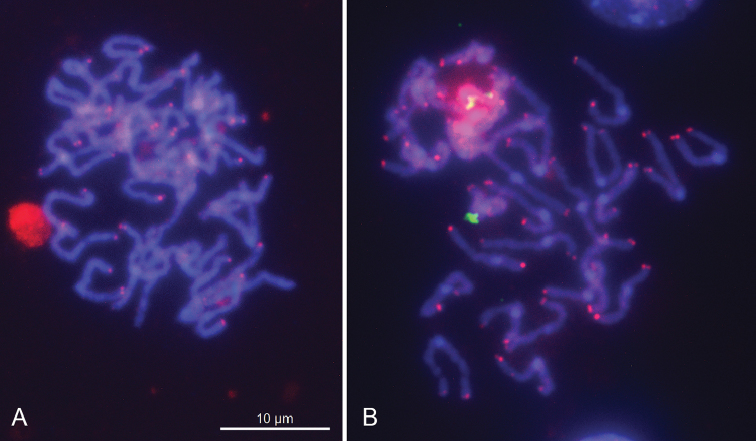
FISH with the “vertebrate” (TTAGGG)_n_ telomeric probe (red signals) on mitotic chromosomes of *H.oxycnida* (**A**) and *P.oligactis* (**B**). The chromosomes are counterstained with DAPI.

**Figure 3.**
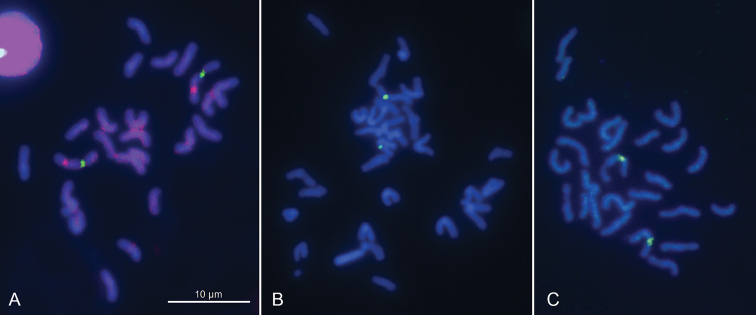
FISH with the 18S rDNA (green signals) and H3 histone (red signals) probes on mitotic chromosomes of *Hydramagnipapillata* (**A**), and with the 18S rDNA probe only on mitotic chromosomes of *Hydraoxycnida* (**B**) and *Pelmatohydraoligactis* (**C**). In *H.magnipapillata*, the FISH signals derived from the 18S and H3 probes are visible on the largest pair of chromosomes, on their long arms and in the centromeric areas respectively. Chromosomes are counterstained with DAPI.

Cytogenetic analyses were carried out on 10 specimens of every species (asexual forms), *Hydramagnipapillata*, *H.oxycnida*, and *P.oligactis*. Representative mitotic images of the species subjected to routine chromosome staining, C-banding, and FISH with the 18S rDNA, histone H3 and telomere (TTAGGG)*_n_* probes are shown in Figures 1–3.

### *Hydra magnipapillata*

The karyotype was found to consist of 30 m/sm chromosomes (2n = 30), it is symmetrical in structure, with chromosomes showing a regular gradation in size. No heteromorphic chromosome pair (putative sex chromosomes) is identified. The homologues of the largest pair carry achromatic gaps on their long arms. C-banding procedure revealed blocks of constitutive heterochromatin (C-blocks) localized in the centromere areas of the chromosomes (Fig. 1 A, D). FISH mapping of the 18S rDNA and histone H3 probes revealed hybridization signals on the largest pair of autosomes, on their long arms and around the centromeres respectively (Fig. 3A). The rDNA signals position corresponds to that of achromatic gaps, that’s to be expected (Fig. 1 A, D).

### *Hydra oxycnida*

As with *H.magnipapillata*, this species has 2n = 30; its karyotype is symmetrical in structure, with chromosomes showing a regular gradation in size, and no heteromorphic chromosome pair is observed. One of the largest chromosome pairs (the largest or the second largest) carries secondary constrictions on the long arm of every homologue (Fig. 1 B, E). Furthermore, the 18S rDNA signals were detected on the long arms of one of largest chromosome pairs (Fig. 3 B). Again, as in the routinely stained preparations, more precise identification of this pair, whether it is the largest or the second largest one, appeared to be difficult. The (TTAGGG)*_n_* probe hybridized to the termini of every chromosome suggesting this sequence to be characteristic of the species (Fig. 2 A).

### *Pelmatohydra oligactis*

As with both above-mentioned species, this species has 2n = 30; its karyotype is symmetrical in structure, with chromosomes showing a regular gradation in size, and no heteromorphic chromosome pair is observed. C-banding procedure followed by DAPI staining revealed C-blocks in the centromere regions of the chromosomes. All but one chromosome pairs were found to be m/sm. The exception was the smallest pair of chromosomes with very short arms which can be preliminarily identified as a subtelocentric/acrocentric pair (st/a). One of the largest chromosome pairs (the largest but maybe the second largest one) carries secondary constrictions on the long arm of every homologue (Fig. 1 C, F). Furthermore, the 18S rDNA signals were detected on the long arms of one of largest chromosome pairs (Fig. 3 C). Again, as in the routinely stained preparations, more precise identification of this pair, whether it is the largest or the second largest one, appeared to be difficult. The (TTAGGG)*_n_* probe hybridized to the termini of every chromosome suggesting this sequence to be characteristic of the species (Fig. 2 B).

## Discussion

### Characterization of karyotypes using standard staining and C-banding technique

Basic features of karyotypes revealed here in *Hydramagnipapillata*, *H.oxycnida*, and *Pelmatohydraoligactis* agree with those reported for these species previously (Anokhin and Kuznetsova 1999, Anokhin and Nokkala 2004, Anokhin et al. 2010). All hydra species studied so far have 2n = 30 with chromosomes showing a regular gradation in size, suggesting thus these features are under stabilizing natural selection. Among chromosomes, there is no pair to be taken as that of sex chromosomes. The centromere position is generally difficult to distinguish after conventional staining, and only C-banding is able to solve this question since C-heterochromatin in the hydra chromosomes is invariably located in the centromere regions (Anokhin and Nokkala 2004, Zacharias et al. 2004, present paper). The karyotypes of *H.magnipapillata* and *P.oligactis* as well as karyotypes of previously studied *H.circumcincta* Schulze, 2014 and *H.vulgaris* Pallas, 1766 (Anokhin and Nokkala 2004) are symmetrical and consist of mainly m/sm chromosomes. At the same time, a comparison between C-banded karyotypes of *P.oligactis* and *H.magnipapillata* showed that the former species had two subtelo/acrocentric (st/a) chromosomes, whereas the last-mentioned species had m/sm chromosomes only. This observation makes it apparent that some chromosome rearrangements have occurred during hydra species evolution, and thus, the species with the same chromosome number can differ one from another in chromosome morphology. The resolving of the issue needs to study in depth.

### Characterization of karyotypes using FISH with the “vertebrate” (TTAGGG)_n_ telomeric probe

Previous studies on *Hydravulgaris* (Traut et al. 2007) and *H.magnipapillata* (Anokhin et al. 2010) have shown that these species possess the “vertebrate” (TTAGGG)_n_ motif of telomeres. Our FISH analyses also showed the presence of this motif at the ends of chromosomes of *H.oxycnida* and *Pelmatohydraoligactis*. Furthermore, the “vertebrate” telomeric sequence is present in representatives of all basal metazoan groups (Traut et al. 2007) and, with some notable exceptions (nematodes and arthropods), is conserved in most Metazoa. Bearing in mind that the “vertebrate” TTAGGG telomeric repeat is widely distributed and is present in most major eukaryotic groups, it is assumed to be the ancestral motif of telomeres in eukaryotes as a whole (Traut et al. 2007, Gomes et al. 2010, Fulnečková et al. 2013).

### Characterization of karyotypes using FISH with 18S rDNA and H3 probes

The chromosomal location of the 18S rRNA genes was studied here in all three species. *Hydramagnipapillata* was shown to have 18S rDNA sites on the large arms of the largest chromosome pair. In *H.oxycnida* and *Pelmatohydraoligactis*, these sites were revealed on one of the largest pairs, the largest or maybe on the second largest one. In every case, the location of these sites coincides with the achromatic gaps, which are generally referred to as secondary constrictions, the nucleolus organizer region (NOR) involved in the formation of nucleolus (McStay 2016). The chromosomal location of the histone H3 gene family was studied in *H.magnipapillata* only. Noteworthy that mapping of H3 has been achieved for the first time in hydras. *H.magnipapillata* showed the H3 sites in the centromeric areas of the largest pair of chromosomes. It is the species that has received the most study by FISH to investigate the chromosomal distribution of different genes and sequences including genes coding for 18S rRNA and 28S rRNA, a head-specific gene ks1, a gene family DMRT suggested to be involved in sex determination and *Tol2*- like transposable element (Anokhin et al. 2010). The rRNA genes were shown to be co-localized on the homologues of the largest pair of chromosomes, on their long arms. A sex-related gene DMRT was revealed on a pair of chromosomes suggesting thus that it is a dose-regulated sex-determining gene in hydras. Probes specific for the ks1 hybridized to three distinct chromosome pairs, and multiple copies of a Tol2 transposable element gene were found on every chromosome. We have shown here that the major rDNA and the H3 genes are positioned on the same pair of chromosomes of *H.magnipapillata*, on their long arms and in the centromeres respectively, and should be thus inherited together. Furthermore, our results suggest that, in *H.magnipapillata*, the canonical histone H3 appears in the form of its centromere-specific variant CENH3, which is known to be the key histone component of the centromere in eukaryotes (Malik et al. 2002, Black and Bassett 2008).

In conclusion, this study delivers insight into the organization of genomes of hydras by reporting first data on (1) *the chromosomal location* of the H3 histone genes by the example of *Hydramagnipapillata*; (2) the telomere motif and the distribution of the 18S rRNA genes on chromosomes of *Hydraoxycnida* and *Pelmatohydraoligactis*. Our results provide a foundation for further studying the mechanisms involved in the chromosome evolution of this phylogenetically important group having an ancient origin within Metazoa.
